# Random Whole Body Vibration over 5 Weeks Leads to Effects Similar to Placebo: A Controlled Study in Parkinson's Disease

**DOI:** 10.1155/2014/386495

**Published:** 2014-10-13

**Authors:** Heiko Gaßner, Annette Janzen, Ansgar Schwirtz, Petra Jansen

**Affiliations:** ^1^Department of Sport Science, University of Regensburg, 93053 Regensburg, Germany; ^2^Department of Neurology, University of Regensburg, 93053 Regensburg, Germany; ^3^Department of Sport Biomechanics, Technical University of Munich, 80992 Munich, Germany

## Abstract

*Background*. Random whole body vibration (WBV) training leads to beneficial short-term effects in patients with Parkinson's disease (PD). However, the effect of WBV lasting several weeks is not clear.* Objectives*. The aim of this study was to assess a random WBV training over 5 weeks in PD.* Methods.* Twenty-one participants with PD were allocated to either an experimental or a placebo group matched by age, gender, and Hoehn&Yahr stage. The WBV training consisted of 5 series, 60 s each. In the placebo group, vibration was simulated. The primary outcome was the change of performance in Functional reach test (FRT), step-walk-turn task, biomechanical Gait Analysis, Timed up and go test (TUG), and one leg stance.* Findings*. In most of the parameters, there was no significant interaction of “time∗group.” Both groups improved significantly in Gait parameters, TUG, and one leg stance. Only in the FRT [*F*(1,15) = 8.397; *P* < 0.05] and in the TUG [*F*(1,15) = 4.971; *P* < 0.05] the experimental group performed significantly better than the placebo group.* Conclusions*. Random WBV training over 5 weeks seems to be less effective than reported in previous studies performing short-term training. The slight improvements in the FRT and TUG are not clinically relevant.

## 1. Introduction

One of the cardinal symptoms of Parkinson's disease (PD) is postural instability which a lot of patients suffer from. While drugs can improve the other cardinal symptoms (bradykinesia, tremor, and rigor), drugs show no effect or even a detrimental effect on postural instability [1–4]. Therefore, it is essential to consider other treatment methods. Balance training and whole body vibration (WBV) training are two possibilities to counteract postural instability. However, the underlying mechanisms of WBV are not clearly understood and clinical efficacy of WBV in neuromuscular diseases is not proven [5, 6]. Previous studies of the effect on WBV training have shown inconsistent results [7–15].

On the one hand, results have shown that, directly after a random WBV intervention, the symptoms rigor, tremor, and postural stability have been improved in patients with PD [8–10]. Random WBV seems to improve tactile sensitivity better than steady vibrations [11]. Deficits in proprioceptive processes have been reported in PD [12]; therefore, these patients could especially benefit from random WBV. Furthermore, in other neurological diseases like multiple sklerosis, positive effects could be shown with WBV [13].

On the other hand, there was no superior efficiacy of WBV training lasting several weeks in comparison to conventional balance training [14]. In this study with PD patients, steady WBV was used. Also, Arias et al. [15] showed that steady WBV and a placebo treatment lasting several weeks caused the same positive effects in PD patients. Both groups performed their training in 12 sessions on nonconsecutive days over 5 weeks. However, in this study, random vibration was not used.

So far, either immediate effects of random WBV or training with steady WBV lasting several weeks has been evaluated, but not whether there are higher effects of a random WBV training lasting several weeks in comparison to a placebo treatment. Quantitative differences in the performance of tasks with potential fall risk [16, 17] (bending forward, climbing steps) have been shown between participants with PD and healthy controls [18]. Therefore, in this study, the same tasks were assessed to investigate the effect of random WBV in PD during performing these tasks. The aim of this study was to evaluate a training of random WBV lasting 5 weeks with quantitatively measured tasks in comparison to a placebo group to whom the vibration was only simulated. Additionally intrasession effects were assessed with the “Timed up and go test” (TUG) and a “one leg stance test.”

## 2. Methods

### 2.1. Participants

**Figure 1:**
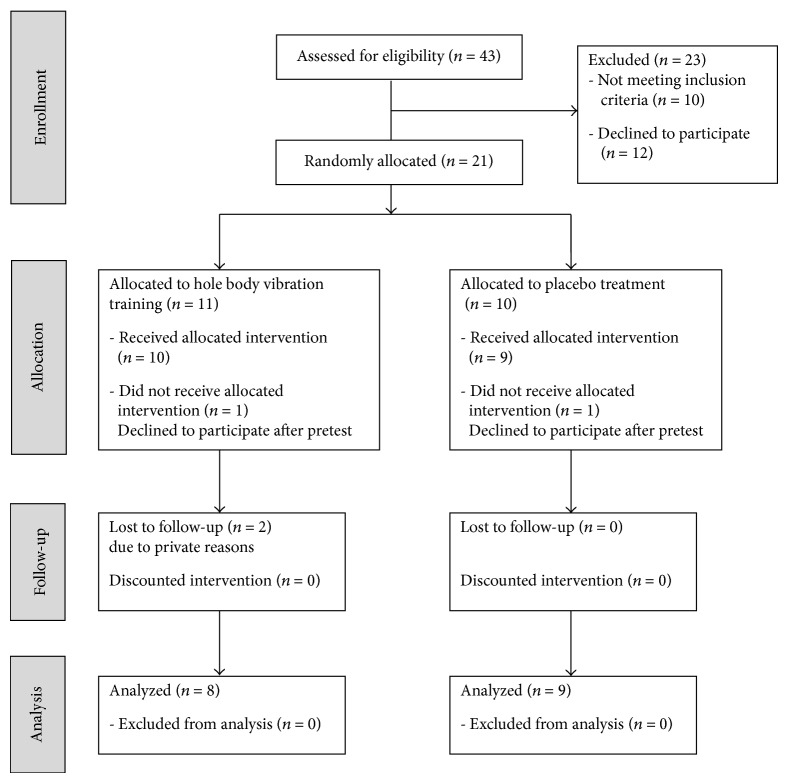
Flow diagram.

In total, 43 participants diagnosed with idiopathic Parkinson's disease (Hoehn and Yahr stage 2-3) were recruited in the outpatient clinic of the Department of Neurology at the University of Regensburg as well as by office-based board certified neurologists in Regensburg. The participants were recruited between July and September 2011, and the trial was performed between October and December 2011. Ten subjects were excluded due to advanced osteoporosis, joint prosthesis, recent lumbar disc hernitation, strong heart beat disturbances, or cancer. Another 12 subjects with PD declined to participate. The remaining 21 participants with PD were matched by age, gender, and the Hoehn and Yahr stage. Afterwards, the pairs were allocated in the experimental group (*n* = 11) and in the placebo group (*n* = 10). In each group, 1 participant declined to continue after pretest and 2 participants of the experimental group did not complete the study due to private reasons (Figure 1). In the remaining subjects of the experimental group (*n* = 8) and of the placebo group (*n* = 9), there were no significant differences concerning age, height, weight, arm length, leg length, disease duration, or daily equivalent dose of L-dopa (Table 1). Furthermore, dementia could be excluded in all participants (MMSE ≥ 24). All experimental trials as well as the training sessions were performed in the ON-period one hour after medication intake. There was no change of dopaminergic medication during the trial period. The execution of this study was approved by the local ethics committee. All participants gave written informed consent according to the Declaration of Helsinki.

### 2.2. Material

**Figure 2:**
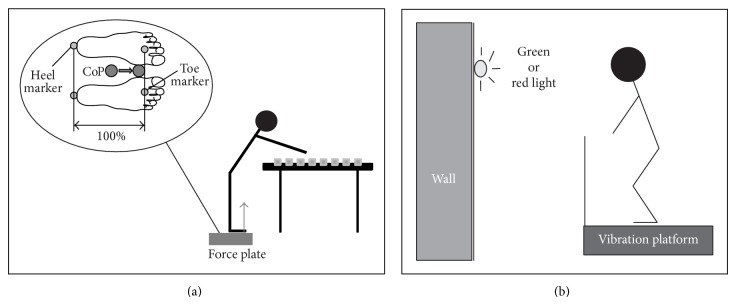
(a) Visualization of the Functional Reach Test (FRT) and calculation of the Center of Pressure (CoP) to the normalized foot length in %. The horizontal distance between heel marker and toe marker was defined as 100%. (b) Test set-up for the intervention. Both groups stood on the vibration platform with knees slightly bend. The placebo group had additionally to concentrate on the light on the wall.

For the assessment of “Gait,” the “Functional Reach Test” (FRT) and the “step-walk-turn task” (see below) kinematics were measured using a six infrared camera motion analysis system (VICON, Oxford Metrics, 200 Hz) and 16 mm passive reflective markers. All markers were placed according to the Plug-in gait full body-model. Ground reaction forces were collected from a three-dimensional force plate (AMTI, OR6-2000, 1000 Hz) and the center of pressure (CoP) was calculated. The CoP was normalized to each participant's foot length. We defined the distance between heel marker and toe marker as 100%. The CoP position concerning each subject's foot length was measured in “standing still” and for the displacement of the CoP during “bending forward” in the FRT. For visualization see Figure 2(a).

### 2.3. Procedure and Measurements

Seven days before the first and after the last training session, tasks of potential fall risk (Gait, Functional reach and Step up and down, as well as turning—evaluated in [18]) were quantitatively assessed and the data of the UPDRS motor score were collected.

#### 2.3.1. Biomechanical Gait Analysis

Instrumented 3D gait analysis was used to quantitatively measure gait parameters (velocity, step length, cadence, double support, and single support) and to describe gait impairments in PD [19]. Participants with PD walked in the laboratory with their self-chosen velocity on a 9 m walkway while kinematic and kinetic data were measured. After habituation, 10 trials were recorded. For data analysis three trials for each leg (strike on the force plate) were taken into account. The mean spatiotemporal parameters and the mean ground reaction forces were analyzed.

#### 2.3.2. Functional Reach Test (FRT)

This common test described by Duncan et al. [20] was slightly modified to reflect activities of daily living measured by motion capture. Participants tried to reach forward as far as possible and grasped a pen with one hand from a table without lifting their heels (table height 0.80 m, distance toe to table 0.30 m). Some pens were distributed across the complete length of the table with an inter-pen-distance of 0.05 m. Each participant performed this task three times with the preferred arm. Mean values of the maximum reach distance and the displacement of the CoP were used for analysis.

#### 2.3.3. Step-Walk-Turn Task

This task was evaluated before and distinguishes patients with PD and healthy controls [18]. Participants must step up onto a 0.25 m high box, walk 2 m to the end of the box, turn 180 degrees, walk back, and step down. The step before stepping up and the step used to get down were placed on a force plate. Ground reaction forces, the resulting velocity of the trunk in stepping down, steps for turning, and the time needed to accomplish the task were measured. The participants performed this task three times and mean values were used for analysis.

#### 2.3.4. UPDRS Motor Score

The UPDRS motor score was assessed by board-certified neurologists who also did a classification of the Hoehn and Yahr stage [21, 22].

Furthermore, acute effects of the intervention were evaluated using the “Timed up and go” test (TUG) and a “one leg stance test,” both explained below. At pretest and posttest as well as on the 3rd, 5th, 7th, 9th, and 11th training sessions, participants had to perform these two tasks (5 intrasession tests).

#### 2.3.5. TUG

Participants sit on a chair, stand up, walk 3 m, turn, walk back to the chair, and sit down [23]. The time to accomplish the task was recorded with a stopwatch.

#### 2.3.6. One Leg Stance Test

Due to reasons of optimal clinical balance assessment, the one leg stance test was performed in addition to the gait and pull test items of the UPDRS [24]. Participants stand unsupported on the preferred leg as long as they can (maximum 60 s). The time in which one leg stance could be performed was recorded with a stopwatch.

### 2.4. Intervention

In the same five-week time period, both groups received an intervention of 12 sessions on nonconsecutive days, two to three times a week. The experimental group exercised on the vibration platform SRT Zeptor Medical plus noise. In order to make an accurate comparison to previous studies [8, 9, 15], 5 sets of stimulation lasting 60 s with a frequency of 6 Hz (±1 Hz noise, 3 mm vibration amplitude) were performed by the experimental group. The break between the sets was set to 60 s. During the intervention, the participants were instructed to stand on the platform in a double supported stable stance with their knees slightly bent.

The placebo group, being not aware of the common vibration stimulus (single-blinded), stood on the vibration platform in the same basic position as the experimental group. The participants of the placebo group were instructed to stand as still as possible during the intervention with the target of keeping body sway to a minimum. In the placebo group the vibration was just simulated by an audible and noticeable signal. A small vibration device was put on the body of the vibration platform and could be switched on or off by a remote control. The vibration feels as if someone is using a percussion drill against the floor next door. However, the vibration of the small device was not transferred to the vibration platform. In contrast to the experimental group, participants of the placebo group had to additionally draw attention to a visual signal fixed on the wall. As long as participants stood still the light was green. When their body began to sway the examiner used another remote control to switch the green light to red which signaled the participants to concentrate on standing still (Figure 2(b)). The participants thought that the visual feedback was connected to the vibration platform.

### 2.5. Statistical Analysis

All statistical analyses were performed using PASW Statistics for Windows, Version 18.0.0. Anthropometric differences between groups were tested by the *t*-test for independent samples. The significance level was set at *P* < 0.05. To calculate the effects of the intervention a 2 × 2 ANOVA with repeated measures was used with “group” (experimental-placebo) as the between subject factor and “time” (pre-post) as the within subject factor. To investigate possible short term effects, an ANOVA with repeated measures was conducted and “group” was set as the between subject factor. The within subject factor “time” was defined with 7 levels (pretest, Session 3, 5, 7, 9, and 11, and posttest). The difference between the current test and the previous test was statistically assessed. For post hoc tests, a Bonferroni alpha-correction was performed.

## 3. Results

There was a significant main effect of factor “time” (pre-post) in some variables. All gait parameters changed significantly in both groups from pretest to posttest: velocity (*F*(1,15) = 28.785; *P* = 0.000; *η* ^2^ = 0.657 [*η* ^2^ = partial eta-quadrat]), step length (*F*(1,15) = 5.740; *P* = 0.030; *η* ^2^ = 0.277), and cadence increased (*F*(1,15) = 32.234; *P* = 0.000; *η* ^2^ = 0.682), double support (*F*(1,15) = 33.037; *P* = 0.000; *η* ^2^ = 0.688) and single support decreased (*F*(1,15) = 18.870; *P* = 0.000; *η* ^2^ = 0.557). In the step-walk-turn task a significant main effect of factor “time” was seen in steps for turning (*F*(1,15) = 5.310; *P* = 0.036; *η* ^2^ = 0.261) and in the time to accomplish the task (*F*(1,15) = 18.441; *P* = 0.001; *η* ^2^ = 0.551). A significant main effect of factor “time” was also seen in the TUG (*F*(1,15) = 12.422; *P* = 0.003; *η* ^2^ = 0.453) and the one leg stance test (*F*(1,15) = 7.779; *P* = 0.014; *η* ^2^ = 0.341).

There were no significant main effects of the factor “group” in any of the measured parameters.

The main outcome of this study is the interaction between “time” (pre-post) and “group” (experimental-placebo). Only in a few variables a significant interaction could be found: reach distance in the FRT (*F*(1,15) = 11.878; *P* = 0.004; *η* ^2^ = 0.359), TUG (*F*(1,15) = 4.971; *P* = 0.041; *η* ^2^ = 0.249), and UPDRS Item “Rigidity” (*F*(1,15) = 6.281; *P* = 0.024; *η* ^2^ = 0.295). In the FRT and TUG the experimental group performed better in the posttest whereas in the “Rigidity” Item the placebo group improved lightly from pretest to posttest. All other variables showed no significant interaction of “time∗group” (Table 2).

### 3.1. Intrasession Evaluation

The intrasession evaluation of the TUG showed a significant main effect of the factor “time” at the 5th, 7th, 9th, and 11th sessions and posttest, respectively, in comparison to the previous session (all *P* values < 0.05). There were significant interactions of “time∗group” between the 5th and 3rd sessions (*F*(1,15) = 8.371; *P* = 0.011; *η* ^2^ = 0.358). The experimental group [mean (SD): 5th session: 9,8 (2,4) s; 3rd session: 11,0 (2,4) s] improved significantly but the placebo group [5th session: 11,1 (2,2) s; 3rd session: 10,8 (2,8) s] did not change TUG performance. Between the 11th and 9th session there was a significant interaction (*F*(1,15) = 5.461; *P* = 0.034; *η* ^2^ = 0.267) but single comparison did not reach significance. There was no significant main effect of the factor “group” (*F*(1,15) = 0.282; *P* = 0.603).

**Figure 3:**
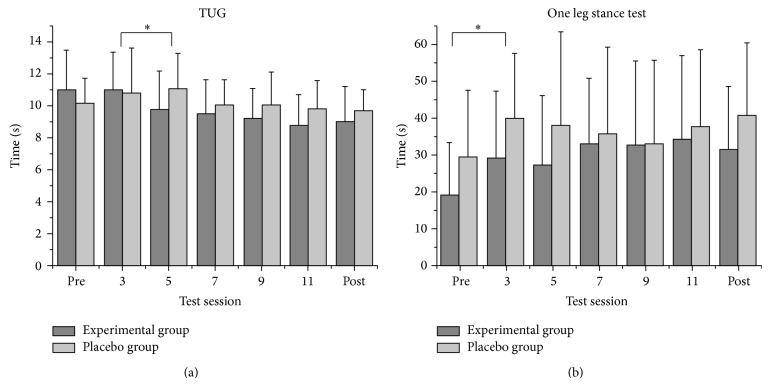
Results of the intrasession evaluation in the “Timed up and go test” (TUG (a)) and the “one leg stance test” (b). TUG: significant interaction “time∗group”; one leg stance: significant main effect of factor “time” (^*^ *P* < 0.05).

For the “one leg stance,” there was a significant main effect of the factor “time” between the 3rd session and pretest (*F*(1,15) = 4.888; *P* = 0.043; *η* ^2^ = 0.246) (Figure 3). Between all other test sessions, there were no significant main effects of the factor “time.” There were also no significant interactions of “time∗group” between the different test sessions and the main effect of the factor “group” was not significant.

## 4. Discussion

The aim of this study was to evaluate an intervention using random WBV lasting 5 weeks with quantitatively measured tasks in comparison to a placebo group.

In most of the parameters a significant interaction of the main outcome measure “time∗group” could not be established. Significant differences could just be found in the reach distance, TUG, and “Rigidity” Item of the UPDRS. All other measured parameters improved in both groups or did not change significantly from pretest to posttest. In contrast to other studies [14, 15], we used random WBV but the effect of the experimental group was similar to the placebo group. Analogical results have been shown in a previous study which used a steady vibration stimulus and equivalent training conditions as in this investigation [15]. Therefore, we hypothesize that random WBV will not lead to more benefit than a steady WBV when training consists of 12 sessions in a period of 5 weeks with the training conditions used in previous studies [8, 9, 15].

In the past, Griffin [25] has shown that organisms have a high sensitivity to vibration at 4–8 Hz whereas vibration frequencies above 10 Hz are less important. The frequency of 6 Hz fits within the suggested range but it is possible that other frequencies in the range of 4–8 Hz are more effective. In this context, a recent study including subjects with PD showed no significant short time effects of WBV between the frequencies 3 Hz, 6 Hz, and 9 Hz and in comparison to a placebo group [26]. These results support the demand for further investigation of different vibration frequencies. Additionally, the necessary number of WBV training sessions should be examined in the future because subjects with PD who participated in our study reported that the main training effects only lasted for several hours. This statement confirms the results of a recent review which concludes that WBV leads to short term improvements in PD whereas longer duration of WBV has no greater beneficial effect in comparison to physical therapy [27].

As King et al. [10] established, there was an increase in step length as well as a decrease in rigidity and tremor when treated by short-term WBV in comparison to a control group which did not get additional treatment. In this study, the placebo group also improved in some parameters from pretest to posttest and performed better than the experimental group, especially in the “Rigidity” Item of the UPDRS Motor Score. The impact of the placebo treatment could explain the improvement of rigidity in the placebo group, because in previous studies it has been shown that the placebo effect wields more influence on bradykinesia and rigor than on tremor, gait, and balance [28]. However, the placebo group also improved in gait; therefore, other explanations have to be taken into account. In previous studies, it has been shown that acoustic signals can improve motor symptoms in participants with PD like arm and finger movements [29] or gait parameters [30, 31]. Also visual feedback has been shown to improve balance [32] and gait [33] in PD. Therefore, it is conceivable that the positive effects in gait parameters arose from acoustic signals or visual feedback, both used in the placebo treatment. Moreover, the frequent testing of the TUG and the “one leg stance” during the training period could have had an effect on gait and as well as on turning. We assume that the placebo group could have been improved in some parameters due to different reasons. However, we have shown that random WBV training in the training conditions we employed is not more effective than a placebo treatment, which additionally included acoustic signals and visual feedback.

Although there was a significant interaction of “time∗group” in the reach distance of the FRT and TUG, prepost differences were not huge between groups. The effect sizes of both parameters were weak, suggesting that random WBV has caused differences of scientific relevance than of clinical relevance in this study. Interestingly, both groups improved their performance in the one leg stance by about 10 s (30%) over five weeks. This improvement probably could be more an effect of frequent task repetition and habituation than of the WBV or placebo intervention. That means also the changes in the FRT and TUG could be established due to motor task learning while performing repeated measures. Taken together, it is unlikely that the random WBV intervention is responsible for functional improvements in PD after a 5 week training period.

## 5. Limitations

The results of this study are limited by the sample size. Furthermore, subjects with different forms (acinetic-rigid, tremor dominant) of PD participated and both the training sessions and the tests were performed in the ON period.

## 6. Conclusion

In this study, we showed that an intervention with random WBV could lead to effects similar to a placebo treatment. Regarding a training over 5 weeks, the random WBV stimulus seems to be less effective than expected in studies which investigated short-term effects. The stimulus type (random/steady) requires further investigation in the future, in addition to the vibration frequency, training position, and the number of the vibration sessions.

## Figures and Tables

**Table 1:** Anthropometric and clinical data of participants.

	Group	Sex	Age (a)	Height (m)	Weight (kg)	Body mass index (kg/m^2^)	Arm length (m)	Leg length (m)	Foot length (m)	Disease duration (a)	Hoehn and Yahr (stage)	L-Dopa equivalent dose (mg/day)
1	Experimental	m	74	1.83	80.0	23.9	0.87	0.96	0.30	8	2.0	0
2	Experimental	m	76	1.75	86.9	28.4	0.86	0.89	0.27	5	2.5	600
3	Experimental	f	75	1.62	66.3	25.3	0.78	0.78	0.26	4	3.0	0
4	Experimental	m	70	1.69	73.8	25.8	0.76	0.82	0.25	10	3.0	400
5	Experimental	m	66	1.75	100.1	32.7	0.79	0.85	0.28	15	2.5	700
6	Experimental	f	68	1.56	48.1	19.8	0.75	0.85	0.27	10	3.0	700
7	Experimental	m	76	1.71	87.7	30.0	0.82	0.84	0.27	2	3.0	112.5
8	Experimental	m	66	1.68	68.2	24.2	0.88	0.84	0.27	7	2.5	400

Mean (SD)			71.4 (4.4)	1.70 (0.08)	76.4 (16.1)	26.3 (4.0)	0.81 (0.05)	0.85 (0.05)	0.27 (0.02)	7.6 (4.1)	2.7 (0.37)	364 (296)

9	Placebo	m	70	1.78	70.5	22.3	0.83	0.90	0.27	3	2.5	150
10	Placebo	f	73	1.56	78.1	32.1	0.73	0.76	0.24	5	2.0	700
11	Placebo	m	69	1.62	75.0	28.6	0.76	0.79	0.27	4	2.0	75
12	Placebo	m	73	1.71	79.2	27.1	0.79	0.84	0.28	20	2.5	600
13	Placebo	f	67	1.54	47.8	20.2	0.74	0.79	0.25	2	3.0	150
14	Placebo	m	74	1.69	60.3	21.1	0.79	0.91	0.27	4	3.0	400
15	Placebo	m	60	1.65	63.9	23.5	0.86	0.88	0.26	8	2.5	600
16	Placebo	m	62	1.86	85.2	24.6	0.87	0.95	0.30	17	3.0	925
17	Placebo	m	66	1.68	68.2	24.2	0.88	0.84	0.27	7	2.5	400

Mean (SD)			68.2 (4.9)	1.68 (0.10)	69.8 (11.4)	24.9 (3.8)	0.81 (0.06)	0.85 (0.06)	0.26 (0.02)	7.8 (6.4)	2.6 (0.39)	444 (287)

**Table 2:** Statistical differences between pretest and posttest (FRT = Functional Reach Test, CoP = Center of Pressure, TUG = Timed up and go test, and UPDRS = Unified Parkinson's Disease Rating Scale).

		Experimental group				Placebo group				Interaction “time∗group”
		Pretest		Posttest		Pretest		Posttest	
		Mean (SD)	95% CI	Mean (SD)	95% CI	Mean (SD)	95% CI	Mean (SD)	95% CI
FRT	Reach distance (m)	**0.89** (7.4)	0.83–0.95	**0.92** (8.4)	0.85–0.99	**0.91** (3.9)	0.88–0.94	**0.89** (3.9)	0.86–0.93	***P* = 0.004**
	CoP (%) standing still	**55.1** (5.8)	50.3–60.0	**55.8** (4.1)	52.4–59.2	**58.5** (8.8)	51.7–65.3	**57.5** (9.2)	50.5–64.6	*P* = 0.538
	CoP (%) reaching forward	**103.9** (7.3)	97.7–110.0	**106.6** (4.1)	103.1–110.0	**107.8** (5.5)	103.5–112.1	**107.8** (6.3)	103.0–112.7	*P* = 0.360

Step-walk-turn task	Resulting ground reaction force (N/kg) in stepping down	**17.2** (3.2)	14.6–19.9	**19.2** (7.1)	13.3–25.0	**19.3** (6.1)	14.6–24.0	**21.3** (6.2)	16.6–26.1	*P* = 0.976
	Resulting velocity of the trunk in stepping down (m/s)	**1.23** (0.12)	1.13–1.32	**1.29** (0.15)	1.16–1.42	**1.40** (0.23)	1.23–1.58	**1.41** (0.19)	1.26–1.55	*P* = 0.418
	Steps for turning	**4.8** (0.7)	4.2–5.3	**4.0** (0.9)	3.2–4.7	**4.4** (1.8)	3.1–5.8	**4.2** (1.4)	3.1–5.3	*P* = 0.201
	Time to accomplish the task (s)	**8.85** (1.22)	7.83–9.87	**7.45** (1.51)	6.19–8.71	**8.03** (1.75)	6.69–9.38	**7.29** (1.67)	6.00–8.58	*P* = 0.209

Gait	Velocity (m/s)	**1.05** (0.10)	0.97–1.13	**1.17** (0.14)	1.05–1.29	**1.11** (0.14)	1.00–1.22	**1.20** (0.13)	1.10–1.31	*P* = 0.416
	Step length (m)	**0.58** (0.06)	0.53–0.63	**0.61** (0.09)	0.53–0.69	**0.58** (0.05)	0.55–0.62	**0.60** (0.04)	0.57–0.63	*P* = 0.501
	Cadence (steps/min)	**110** (11.3)	100–119	**118** (15.9)	104–131	**114** (11.8)	105–123	**121** (11.3)	111–129	*P* = 0.679
	Double support (s)	**0.29 **(0.05)	0.24–0.33	**0.25 **(0.05)	0.21–0.29	**0.28** (0.05)	0.24–0.32	**0.24** (0.05)	0.20–0.28	*P* = 0.855
	Single support (s)	**0.42** (0.04)	0.38–0.45	**0.40** (0.05)	0.36–0.44	**0.40** (0.04)	0.37–0.43	**0.38** (0.03)	0.36–0.41	*P* = 0.581

TUG	Time (s)	**11.0** (2.5)	8.9–13.1	**9.0** (2.2)	7.2–10.8	**10.1** (1.6)	8.9–11.4	**9.7** (1.3)	8.7–10.7	***P* = 0.041**

One leg stance	Time (s)	**18.9** (14.4)	6.9–30.9	**31.5** (17.1)	17.2–45.8	**29.4** (18.0)	15.6–43.3	**40.8** (19.6)	25.8–55.8	*P* = 0.822

UPDRS Motor Score	Score	**29** (14)	17–41	**27** (13)	17–38	**19** (7)	14–24	**18** (6)	13–22	*P* = 0.708

Subitems										
Postural stability	Score	**1.4** (0.5)	0.9–1.8	**1.1** (0.4)	0.6–1.5	**0.9** (0.6)	0.5–1.3	**0.7** (0.7)	0.2–1.1	*P* = 0.725
Rigidity	Score	**5.1** (4.1)	2.8–7.5	**5.8** (4.4)	3.3–8.2	**3.3** (1.9)	1.1–5.5	**2.8** (1.6)	0.5–5.1	***P* = 0.024**
Tremor	Score	**2.1** (3.8)	0.5–4.3	**1.4** (2.3)	0.0–2.7	**1.3** (1.7)	0.7–3.3	**1.0** (1.3)	0.3–2.3	*P* = 0.596
Bradykinesia	Score	**1.8** (0.9)	1.0–2.5	**1.8** (0.9)	1.0–2.5	**1.3** (0.5)	1.0–1.7	**1.2** (0.7)	0.7–1.7	*P* = 0.362
